# Unraveling the blue shift in porphyrin fluorescence in glioma: The 620 nm peak and its potential significance in tumor biology

**DOI:** 10.3389/fnins.2023.1261679

**Published:** 2023-11-06

**Authors:** Eric Suero Molina, David Black, Anna Walke, Ghasem Azemi, Fabio D’Alessandro, Simone König, Walter Stummer

**Affiliations:** ^1^Department of Neurosurgery, University Hospital of Münster, Münster, Germany; ^2^Computational NeuroSurgery (CNS) Lab, Macquarie Medical School, Faculty of Medicine, Health and Human Sciences, Macquarie University, Sydney, NSW, Australia; ^3^Department of Electrical and Computer Engineering, University of British Columbia, Vancouver, BC, Canada; ^4^Core Unit Proteomics, Interdisciplinary Centre for Clinical Research, University of Münster, Münster, Germany

**Keywords:** 5-ALA, fluorescence-guided resection, autofluorescence, PpIX photo-states, hyperspectral imaging

## Abstract

In glioma surgery, the low-density infiltration zone of tumors is difficult to detect by any means. While, for instance, 5-aminolevulinic acid (5-ALA)-induced fluorescence is a well-established surgical procedure for maximizing resection of malignant gliomas, a cell density in tumor tissue of 20–30% is needed to observe visual fluorescence. Hyperspectral imaging is a powerful technique for the optical characterization of brain tissue, which accommodates the complex spectral properties of gliomas. Thereby, knowledge about the signal source is essential to generate specific separation (unmixing) procedures for the different spectral characteristics of analytes and estimate compound abundances. It was stated that protoporphyrin IX (PpIX) fluorescence consists mainly of emission peaks at 634 nm (PpIX_634_) and 620 nm (PpIX_620_). However, other members of the substance group of porphyrins fluoresce similarly to PpIX due to their common tetrapyrrole core structure. While the PpIX_634_ signal has reliably been assigned to PpIX, it has not yet been analyzed if PpIX_620_ might result from a different porphyrin rather than being a second photo state of PpIX. We thus reviewed more than 200,000 spectra from various tumors measured in almost 600 biopsies of 130 patients. Insufficient consideration of autofluorescence led to artificial inflation of the PpIX_620_ peak in the past. Recently, five basis spectra (PpIX_634_, PpIX_620_, flavin, lipofuscin, and NADH) were described and incorporated into the analysis algorithm, which allowed more accurate unmixing of spectral abundances. We used the improved algorithm to investigate the PpIX_620_ signal more precisely and investigated coproporphyrin III (CpIII) fluorescence phantoms for spectral unmixing. Our findings show that the PpIX_634_ peak was the primary source of the 5-ALA-induced fluorescence. CpIII had a similar spectral characteristic to PpIX_620_. The supplementation of 5-ALA may trigger the increased production of porphyrins other than PpIX within the heme biosynthesis pathway, including that of CpIII. It is essential to correctly separate autofluorescence from the main PpIX_634_ peak to analyze the fluorescence signal. This article highlights the need for a comprehensive understanding of the spectral complexity in gliomas and suggests less significance of the 620 nm fluorescence peak for PpIX analysis and visualization.

## Introduction

Gliomas, the most common primary brain tumors, are still among the most challenging neuro-oncological diseases to diagnose and treat (Schaff and Mellinghoff, 2023). Surgery is the first step in standard multimodal therapy. By using surgical adjuncts such as 5-aminolevulinic acid (5-ALA), neurosurgeons can maximize tumor resection. The use of 5-ALA is well-established in fluorescence-guided neurosurgery for high-grade glioma (HGG) resection and photodynamic therapy (Stummer et al., 2006; Stummer and Suero Molina, 2017; Schipmann et al., 2020). Resection of the fluorescing tissue will regularly exceed the borders of contrast enhancement in magnetic resonance images (Schucht et al., 2014).

5-ALA is a non-fluorescent and naturally occurring prodrug metabolized by malignant tissue, which induces the accumulation of protoporphyrin IX (PpIX). Current evidence shows that malignant glioma tissue exhibits visual fluorescence upon reaching a threshold of tumor cell density ranging from at least 20 to 30% (Stummer et al., 2017; Suero Molina et al., 2020); below this level, fluorescence visualization is limited. Only about 20% of low-grade gliomas (LGG) display visual fluorescence in surgical microscopy (Jaber et al., 2016), which can be significantly improved when doubling the standard 5-ALA dosage from 20 to 40 mg/k.g. b.w. (Suero Molina et al., 2022).

However, many recurrences occur near resection margins (Petrecca et al., 2013). Therefore, current research is directed toward better visualization of the low-density infiltration zone of HGG and LGG. For this purpose, several research groups apply hyperspectral imaging (HI) to increase the sensitivity in optical visualization of malignant tissue (Bravo et al., 2017; Valdes et al., 2017; Black et al., 2021; Walke et al., 2023). The feature of spatially resolved spectroscopy makes HI a powerful tool in the characterization of tissues, potentially aiding in diagnosis. Fast diagnosis of diseases and surgical aiding have been the most desired application of HI in healthcare so far (Mangotra et al., 2023). Moreover, label-free imaging is under current scientific scrutiny, and although not limited to this technique, HI might be the tool for its realization.

Acquiring spatial and spectral information creates a unique fingerprint for each image pixel. Spectral alterations can then be attributed to different biochemical characteristics. Correctly describing the spectral complexity in gliomas is essential in understanding the processes of 5-ALA uptake and the subsequent induction of heme biosynthesis. HI measures a continuous spectrum within a selected spectral range and allows for spectral characterization of autofluorescence and PpIX fluorescence (Black et al., 2021; Walke et al., 2023).

PpIX exhibits maximum absorption at 405 nm, the Soret band, in the blue light range of the electromagnetic spectrum (Stummer et al., 1998). Characteristically, its fluorescence presents a double emission peak at 634 and 705 nm. The difference in wavelength between absorption and emission is known as the Stokes shift, which permits separation of excitation wavelength and emitted fluorescence via optical filters for optimal visualization of tumors and brain tissue.

A second photo-state of PpIX peaking at 620 nm (PpIX_620_) was proposed as a fluorophore in glioma, contributing to the PpIX fluorescence emission (Montcel et al., 2013). A PpIX_620/634_ ratio was described *in vivo*, which increases toward the infiltration zone and decreases toward the tumor core (Montcel et al., 2013; Alston et al., 2019). Thereby, the tumor microenvironment is of importance; it is believed that pH and the macromolecule concentration influence the PpIX photo-state distribution as observed in solution or tissue-simulating phantoms (Melo and Reisaeter, 1986; Lozovaya et al., 1990; Scolaro et al., 2002; Montcel et al., 2013; Alston et al., 2018).

The extracellular pH can be more acidic in malignant than in non-malignant tissue; the cellular pH gradient is considered to be reduced or, in some cases, even reversed in tumors compared to normal tissue (Gerweck and Seetharaman, 1996). Especially in glioma, alterations of pH are thought to be linked with tumor pathogenicity (Honasoge and Sontheimer, 2013). Thus, pH is an essential factor of the tumor microenvironment and should be considered in spectral imaging approaches.

The two PpIX photo-states are reported to have different quantum yields (Lozovaya et al., 1990; Montcel et al., 2013; Jonin et al., 2020), which is why they should be treated separately in spectral unmixing. Recent characterization of basis spectra improved the spectral unmixing algorithm, allowing for a more precise description of the abundances of the major five basis spectra in glioma tissue (PpIX_620_, PpIX_634_, flavin, lipofuscin, and NADH) (Black et al., 2021). This improves the exploration of the two photo-states of PpIX and the autofluorescence spectra from NADH, flavin (i.e., flavin adenine dinucleotide), and lipofuscin (Black et al., 2021). This analysis is particularly interesting for the 620 nm abundance ([PpIX_620_]), as it is artificially inflated when not unmixed with the five basis spectra (Black et al., 2021). Furthermore, porphyrins fluoresce similarly due to their common tetrapyrrole core structure (Seo et al., 2009; Lang et al., 2015), and it has not been analyzed for glioma, if the PpIX_620_ signal might result from other porphyrins rather than being a second photo-state of PpIX.

By performing pH-dependent measurements in pig brain homogenates and creating fluorescence phantoms with coproporphyrin III (CpIII) using hyperspectral imaging, we aimed to better understand the PpIX_620_ peak in gliomas. Furthermore, we reviewed more than 200,000 spectra acquired from almost 600 human biopsies. This article discusses the spectral characteristics of tumors following 5-ALA administration, focusing on the origin of the PpIX_620_ signal.

## Materials and methods

We retrospectively analyzed spectra of biopsies from previous projects (Kaneko et al., 2019, 2021; Black et al., 2021; Suero Molina et al., 2021, 2022; Walke et al., 2023). Patients harboring distinct tumors who received 5-ALA orally 4 h before induction of anesthesia at a dose of 20 mg/k.g. b.w., were selected (*n* = 130). As varying information was available for the different analyzed criteria, we selected respective subsets of patients for each analysis (Table 1).

**Table 1:** Number of available data for this study (IDH = isocitrate dehydrogenase).

	Patients	Biopsies (B)/Homogenates (H)	Spectra
pH	N/A	40 (H)	65,444
IDH	75	224 (B)	90,649
Visible Fluorescence	84	244 (B)	96,796
5-ALA dose	23	78 (B)	38,683
Margin	27	288 (B)	173,650
Tumor type	113	513 (B)	271,813

### Acquisition and preprocessing

All data were measured *ex vivo* with a previously described widefield hyperspectral microscopy device (Kaneko et al., 2019, 2021; Black et al., 2021; Suero Molina et al., 2021, 2022; Walke et al., 2023). Spectral and spatial data cubes were generated by varying a tunable bandpass filter from 420 nm to 730 nm and capturing a 2048 × 2048 pixel image with a scientific complementary metal oxide semiconductor (sCMOS) monochrome camera every 3 to 5 nm. This procedure was carried out under intense 405 nm illumination, white light, and no illumination. In each case, light was captured through a ZEISS OPMI Pico microscope and filtered to eliminate the reflected blue excitation light but to retain the fluorescence spectrum. Regions of interest were selected manually in every data cube for the targeted tissue. These regions were divided into 10 × 10 squares of pixels and averaged to create one spectrum per square, thus reducing noise. After correcting for dark noise using the un-illuminated images, the white light spectra were used for dual-band normalization of the fluorescence spectra (Valdes et al., 2012). This process removes bias due to inhomogeneous absorption and scattering across the tissue surface.

By manually selecting regions of interest in every biopsy, we confidently removed the background and present only tumor tissue spectra, further reducing noise. Once extracted and normalized, the spectra were unmixed into their constituent parts using non-negative least squares and *a priori* knowledge of the present fluorophores (Black et al., 2021). This assumes that the measured fluorescence spectrum is a linear combination of individual fluorescences. The weighting of each spectrum in the linear combination describes the relative abundance of that fluorophore in the measurement. Converting these abundances given in arbitrary units (a.u.) to a concentration (for example, μg/mL) has previously been challenging (Black et al., 2021; Walke et al., 2023). This was partly due to the spectral complexity of PpIX and the difference in biochemical microenvironment and optical properties between human tissue and the phantom samples used for calibration. As absolute concentration values are ultimately not required in this paper, we present all results as abundances in arbitrary units (a.u.).

### Control experiments with pig brain homogenates

**Figure 1:**
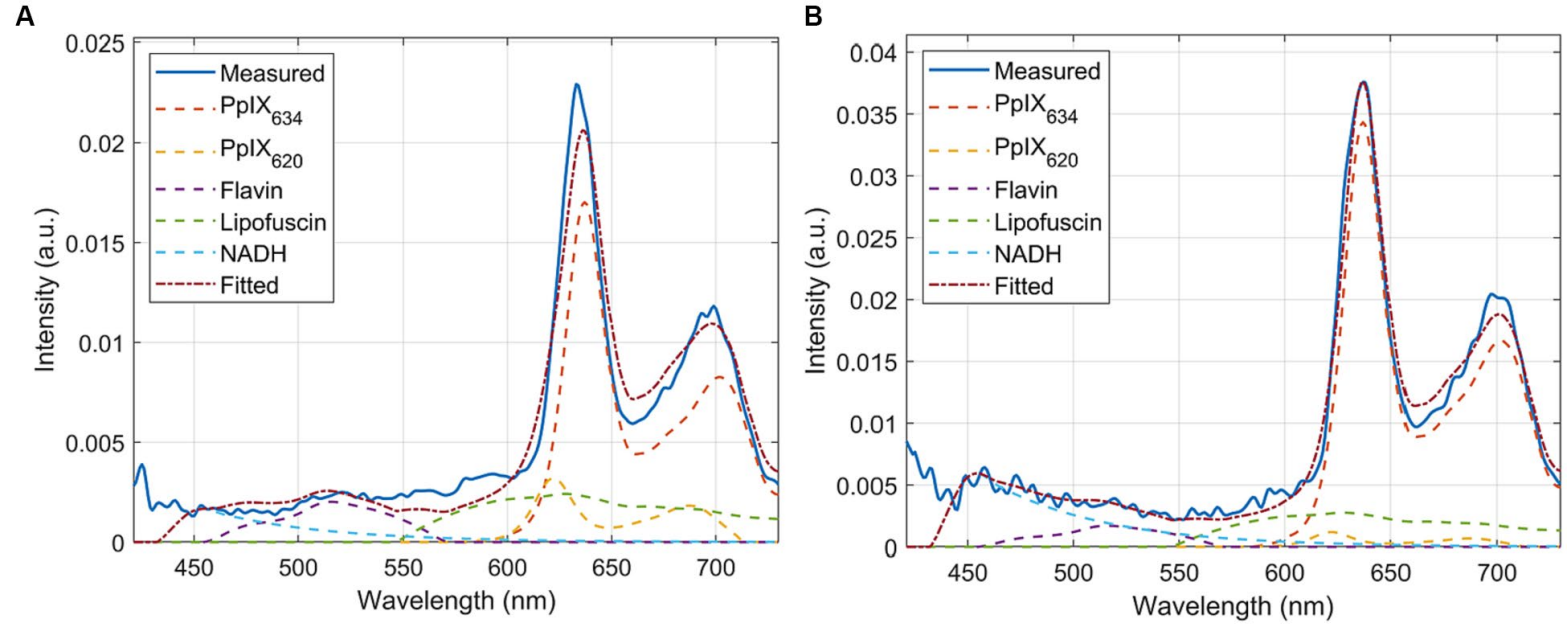
Sample measured spectra and unmixing of **(A)** pig brain homogenates and **(B)** phantoms. These show that the established basis spectra are completely effective on both and are, therefore, representative models of human tissue.

To assess possible spectral alterations related to pH variance, pig brain reference tissue homogenates (RTH), as previously described by Walke et al. (2023) were used. Experiments were permitted by the Health and Veterinary Office Münster (Reg.-No. 055151052 21). Only cerebrum was used for the experiments. Tissue was washed with distilled water, roughly cut into pieces, and homogenized using a blender (VDI 12, VWR International, Hannover, Germany). Adjustment of pH within the range of 5 to 9 was achieved using 0.5 M tris(hydroxymethyl)aminomethane (Tris-base, Serva, Heidelberg, Germany) and hydrochloric acid (HCl, Honeywell Riedel-de Haen, Seelze, Germany). RTHs with pH control (pH-RTHs) were composed of homogenized cerebrum and buffer (w/v) as displayed in Table 2, before adding PpIX stock solution (300 pmol/μL in dimethyl sulfoxide (DMSO), Merck KGaA, Darmstadt, Germany) to the desired concentration of 3.0 pmol/mg. The PpIX concentration was fixed in all evaluated pH-RTHs; only the pH varied (Table 2). An exemplary spectrum is shown in Figure 1A.

**Table 2:** Composition of pH-RTHs.

Tris buffer pH after adjustment with HCl	Final Tris concentration [M]	RTH [mg]	Addition of Tris buffer [μL]	Final pH
5.0	0.5	600	600	5.1*
5.5				5.8*
6.0				6.4*
6.5				6.5
7.0				6.8
7.5				7.2
8.0				7.7
8.5				8.2
9.0				8.8

In samples labeled with *, pH was again adjusted with HCl after mixing Tris buffer with homogenized cerebrum tissue.

### Phantom models

Phantom samples were prepared from RTHs and different porphyrin stock solutions to evaluate the spectral characteristics of various porphyrins. 600 mg cerebrum RTH was directly spiked with PpIX stock solution (300 pmol/μL in DMSO) or CpIII stock solution (300 pmol/μL in DMSO, Livchem Logistics, Frankfurt, Germany), yielding a concentration of 3.0 pmol/mg. pH was not adjusted or controlled in the phantoms. We also intended to test uroporphyrin, which was, however, only available in acidic solution and could thus not be compared to the other porphyrins dissolved in DMSO. PpIX and CpIII phantoms were transferred to a petri dish, forming samples of about 4 × 4 × 2 mm; for measurement of pH-RTHs, black washers were used. PpIX, CpIII phantoms, and pH-RTHs were immediately measured with the same parameters as for clinical biopsy measurements. Measurements were performed with three technical replicates. An exemplary spectrum is shown in Figure 1B.

### Statistical analyses

MATLAB (The Math Works Inc., Natick, Massachusetts) and Python were used for the analysis. Statistical significance was measured using the 2-sample Kolmogorov–Smirnov test or the Wilcoxon rank-sum test, depending on the data type.

### Experiments

The pH effects were studied in pH-RTH, while IDH-mutation (mutated vs. wildtype), fluorescence visibility during surgery (graded as none, weak or strong), 5-ALA dose (20 vs. 40 mg/k.g. b.w), and margin type (solid tumor (ST), infiltration zone (IZ), and reactive altered brain tissue (RABT)) were analyzed in the tumor types shown in Table 3.

**Table 3:** Number of biopsies of different tumor types used in the analysis of each variable.

	IDH mutation	Fluorescence visibility	5-ALA dose	Margin type	Tumor type
	Yes | No	None | Weak | Strong	Single | Double	ST | IZ | RABT	
Pilocytic Astrocytoma	–	3 | 0 | 0	3 | 2	–	5
Diffuse Astrocytoma	52 | 0	39 | 6 | 7	37 | 15	–	52
Anaplastic Astrocytoma	24 | 0	3 | 1 | 20	–	0 | 0 | 10	34
Glioblastoma	0 | 123	11 | 9 | 116	–	124 | 57 | 74	393
Oligodendroglioma	21 | 0	11 | 6 | 4	17 | 4	–	21
Ganglioglioma	–	2 | 2 | 0	–	–	4
Anaplastic Oligodendroglioma	4 | 0	0 | 4 | 0	–	–	4

The numbers result from the availability of the data. Each biopsy yielded, on average, 481 spectra (250 to 740 interquartile range). Respective administrations of a single dose (*n* = 10, 20 mg/kg b.w.) and a double dose (*n* = 13, 40 mg/kg b.w.) of 5-ALA were carried out.

## Results

### pH-variance pig brain homogenates

**Figure 2:**
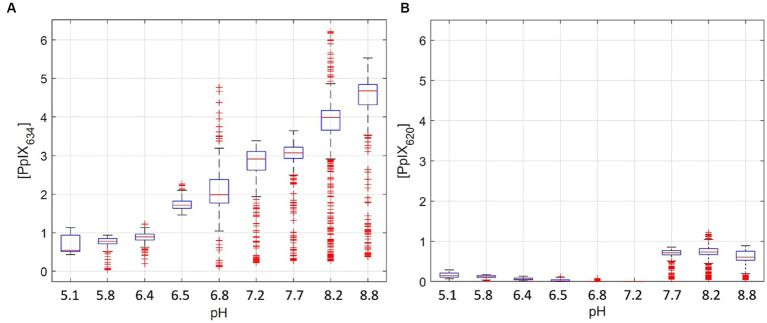
Pig homogenates measurements with 3 pmol/mg PpIX and pH adjustment between 5.1 to 8.8 **(A,B)**. **(A)** shows the [PpIX_634_], and **(B)** shows [PpIX_620_]. The physiological range for normal brain and glioma tissue is between pH 6 and 8 (Gerweck and Seetharaman, 1996; Honasoge and Sontheimer, 2013).

[PpIX_620_] was determined in the RTHs with known pH and PpIX concentration. Using the 2-sample Kolmogorov–Smirnov test, the [PpIX_620_] differed significantly (*p* < 0.001) between different pH levels (Figure 2B). However, unlike [PpIX_634_], [PpIX_620_] is not strongly linearly related to pH (r^2^ = 0.50). For the comparison of the measured [PpIX_634_] values and tissue pH (*p* < 0.001 between all pH values), there is a clear linear trend (r^2^ = 0.88). This is shown in Figure 2 for a given PpIX concentration in cerebrum pH-RTHs. It seems that [PpIX_634_] increases linearly with pH in the tested range (pH 5.1–8.8) while [PpIX_620_] decreases linearly until pH 7.2 and is high in the pH range 7.7–8.8 (Figure 2). Compared to [PpIX_634_], the [PpIX_620_] is lower by about factor five (Figure 2). This shows that PpIX_634_ is more important for PpIX analysis compared to PpIX_620_ when considering pH dependence between pH 5 and 9.

### Human brain data

#### IDH mutation

**Figure 3:**
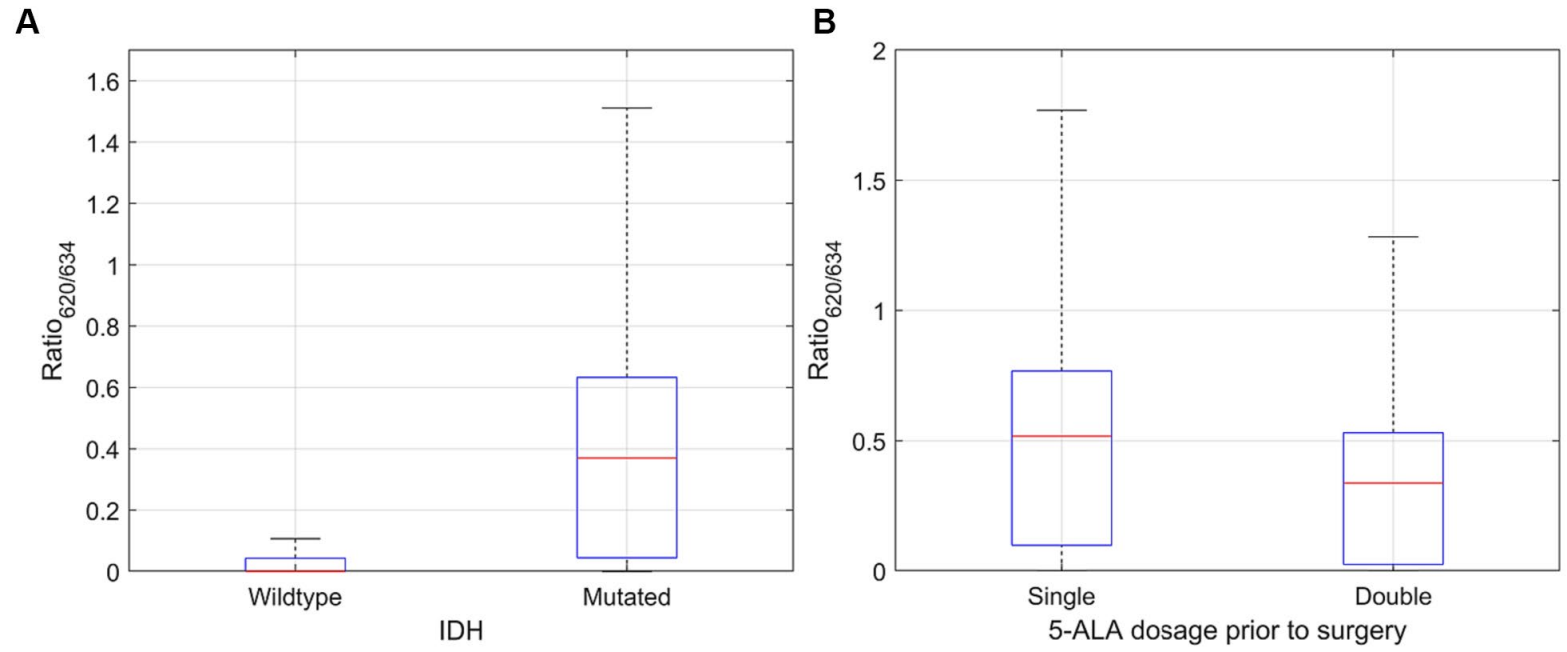
Distribution of [PpIX_620_]/[PpIX_634_] ratio for **(A)** IDH mutated and wildtype glioma (significantly higher for mutated glioma; *p* < 0.001) and **(B)** single (20 mg/k.g. b.w.) and double (40 mg/k.g. b.w.) dose of 5-ALA. The mean and median [PpIX_620_] proportion was significantly higher for single dose (*p* < 0.001).

The abundances for PpIX_634_ and PpIX_620_ were analyzed concerning the IDH mutation in *ex vivo* human tissue samples. IDH mutation was detected for a subset of patients and not for each measured tumor biopsy. The ratio [PpIX_620_]/[PpIX_634_] is much higher for tumors with IDH mutation than in IDH-wildtype tumors. The mean ratio for IDH-wildtype tumors was 0.046 ± 0.168, whereas IDH-mutant tumors presented a 0.477 ± 1.878 mean ratio. The difference is statistically significant with *p* < 0.001. Most of the change in the ratio is due to [PpIX_634_], while [PpIX_620_] does not vary much. Median values are listed in Table 4, and the ratio distributions are shown in Figure 3A. The ratio was calculated for each spectrum, and the mean/median was computed after.

**Table 4:** Median values of [PpIX_620_], [PpIX_634_] and their ratio in IDH-mutated and IDH-wildtype gliomas.

IDH	[PpIX_620_]	[PpIX_634_]	[PpIX_620_] / [PpIX_634_] ratio
Wildtype	0.000	10.052	0.000
Mutant	0.050	0.191	0.370

The ratio was calculated separately for each spectrum, followed by calculation of the median.

#### Double dose of 5-ALA

Doubling the dosage of 5-ALA in LGG leads to a decrease in the median and mean proportion of PpIX_620_. Even though the single-dose group presented some outliers, using the Wilcoxon-Rank-Sum test, a significant difference was observed (*p* < 0.001). The median [PpIX_620_]/[PpIX_634_] ratio after a single (20 mg/k.g. b.w.) vs. a double (40 mg/k.g. b.w.) 5-ALA dose was 0.517 and 0.337, respectively. The mean was likewise higher despite the outliers, with 0.648 ± 2.623 in the single-dose group and 0.346 ± 0.337 in the double-dose group. Figure 3B illustrates the dependence of the ratio [PpIX_620_]/[PpIX_634_] on the 5-ALA dose.

Only 10 patients received a double dose of 5-ALA for a small proof-of-principle study (Suero Molina et al., 2022). Therefore, the subset of patients analyzed in the IDH comparison is much larger. The effect of the double dose in the IDH subset is relatively small.

#### Fluorescence visibility

Before tumor removal and *ex vivo* measurement, the surgeon first graded every brain tumor sample regarding the quality of visible fluorescence. As observed for the pH and IDH, [PpIX_620_] seems far less interesting than [PpIX_634_] concerning fluorescence visibility. The [PpIX_620_] / [PpIX_634_] ratio decreases as visibility increases. This comes, however, from the fact that the [PpIX_634_] increases on average by a factor of 46 while [PpIX_620_] only doubles and is not strongly related to fluorescence visibility (r^2^ = 0.0148), although the categories’ differences are statistically significant (*p* < 0.01). This is shown in Table 5.

**Table 5:** PpIX contributions versus fluorescence visibility.

	[PpIX_634_]	[PpIX_620_]	Ratio
None	0.125; 0.315 ± 0.700	0.050; 0.088 ± 0.050	0.403; 0.543 ± 2.093
Weak	1.061; 2.300 ± 3.179	0.103; 0.136 ± 0.149	0.120; 0.310 ± 0.382
Strong	10.17; 14.34 ± 14.33	0.000; 0.196 ± 0.518	0.000; 0.027 ± 0.151

Values are expressed as median and mean ± standard deviation. The ratio was computed for each spectrum and then averaged.

### Margin classification

**Figure 4:**
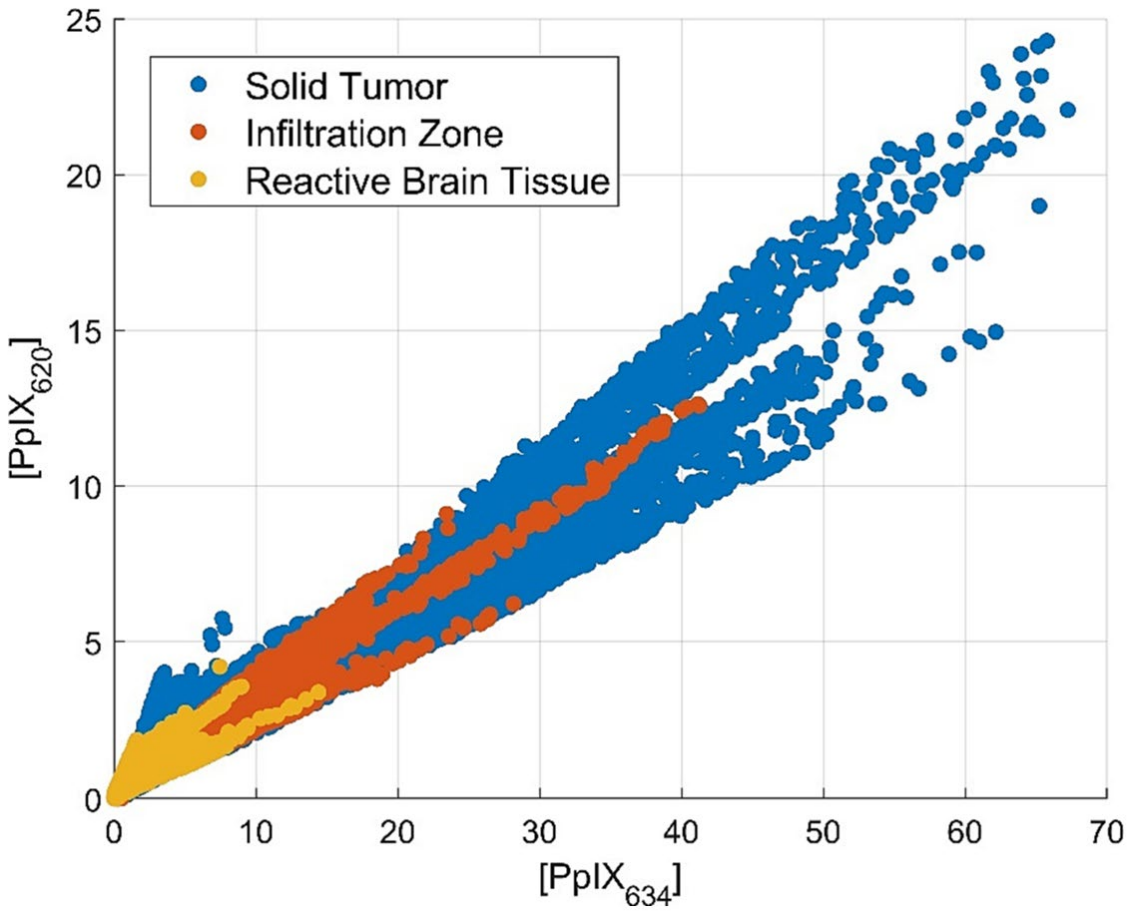
Scatterplot of the PpIX_634_ and PpIX_620_ abundance in solid tumor, infiltration zone, and reactive altered brain tissue showing approximately constant PpIX ratio.

Tissues were divided according to the histopathological assessment between ST, IZ, and RABT. For the comparison of tumor margin and PpIX abundance, both [PpIX_634_] and [PpIX_620_] vary similarly. The abundance is relatively high for ST and decreasing for IZ and RABT. As a result, the ratio does not change as much as in other categories. This is visualized in Figure 4. The mean ratio is 0.472 ± 1.359 (mean ± standard deviation) for ST, 0.907 ± 1.687 (mean ± standard deviation) for IZ, and 1.014 ± 4.286 (mean ± standard deviation) for RABT (median 0.331, 0.635, and 0.705, respectively).

### Phantom experiments

In the various tests described so far, [PpIX_620_] was always relatively constant while [PpIX_634_] varied. If PpIX_620_ would be a second fluorescing state of PpIX, caused by a different microenvironment or pH, as has been hypothesized (Melo and Reisaeter, 1986; Lozovaya et al., 1990; Kim et al., 2010; Montcel et al., 2013; Alston et al., 2019), then one might expect it to vary more. An alternative theory is that the 620 nm peak fluorescence stems from other porphyrin precursors in the heme synthesis pathway, including coproporphyrin III (CpIII) and uroporphyrin (Up) (Dietel et al., 2007; Seo et al., 2009; Lang et al., 2015). We created CpIII and PpIX phantoms to explore this hypothesis, measured them, and extracted 689 CpIII and 1884 PpIX spectra. Up was only available in HCl, which causes a strong peak wavelength shift (Melo and Reisaeter, 1986), thus it could not be used. However, its fluorescence curve is expected to have a PpIX-like shape, peaking at 616 nm (Lang et al., 2015).

Using the pig brain-based phantoms described in the Methods section, the peak wavelengths of these spectra were determined using two methods. First, the maximum of each spectrum was taken and averaged over all the measurements. However, fluorescence is a Poisson process, so the intensity at each wavelength is Poisson distributed rather than Gaussian. Thus, the fluorescence peak maxima are the noisiest regions, and it is easy to miss the maximum by several nanometers. To address this, the spectra were first averaged, which decreases noise by $\sqrt{n}$ . The average spectrum was then fitted to each spectrum in the region of the main peak (from 590 to 640 nm) by maximizing the normalized cross-correlation between the two signals. This fits the peak shape, independent of magnitude.

**Figure 5:**
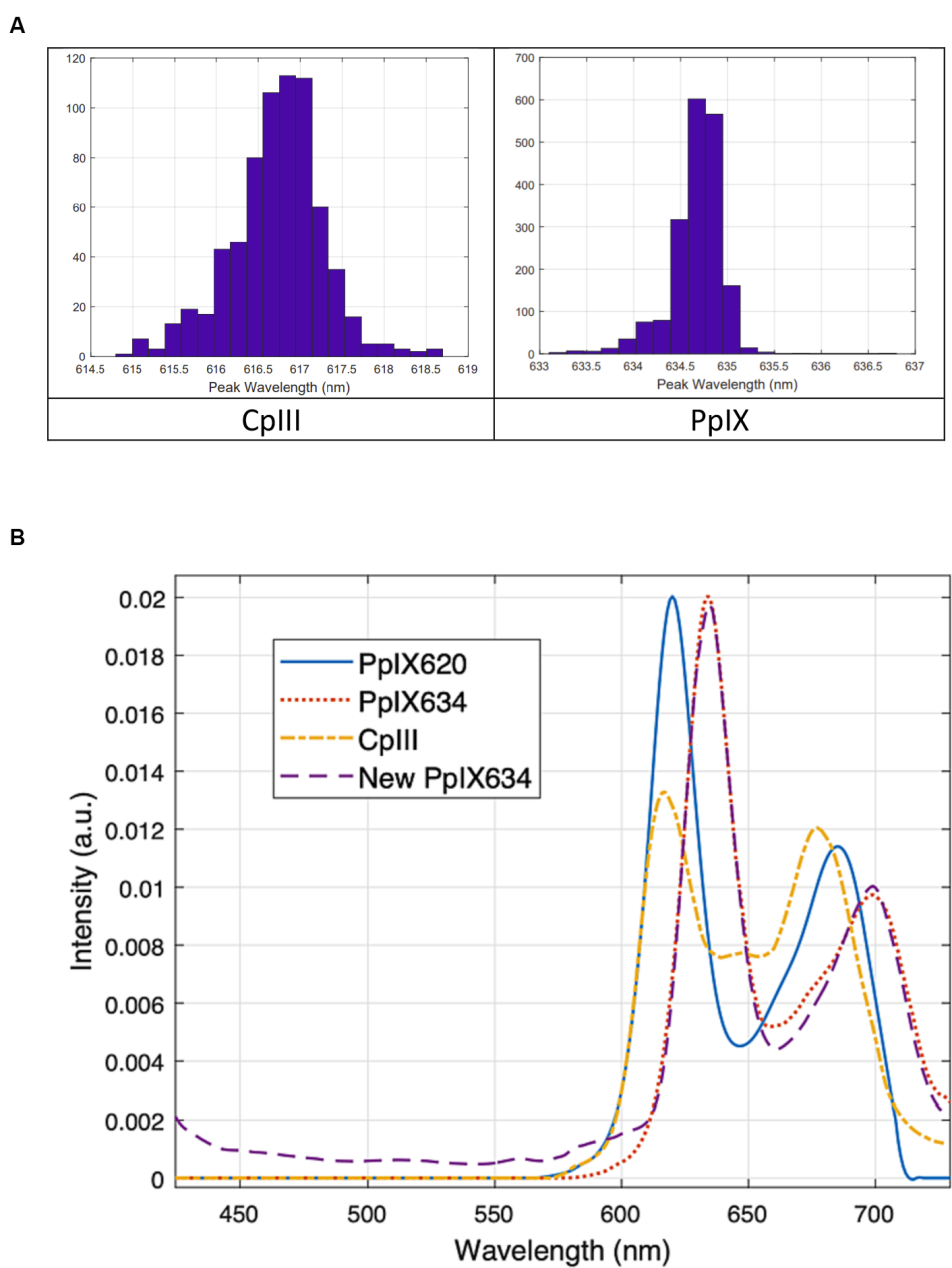
**(A)** Peak location distributions from using the maxima of the individual spectra **(B)** Porphyrin basis spectra for unmixing tests. CpIII and the new PpIX_634_ are based on pig brain phantoms. The old PpIX spectra (labeled PpIX_620_ and PpIX_634_) are from Black et al. (2021). The new and old PpIX_634_ match very well, so the new PpIX_634_ and the CpIII should be directly applicable to our existing human data.

**Figure 6:**
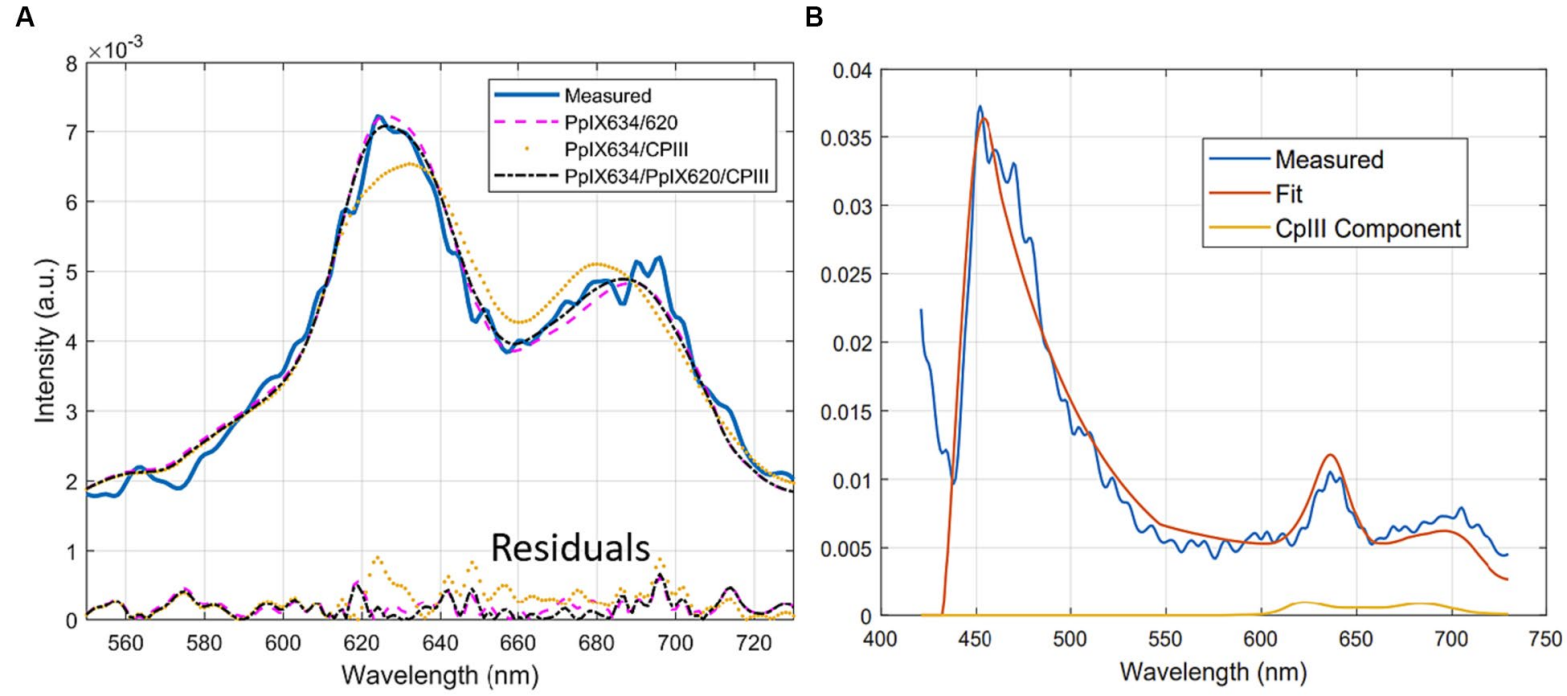
**(A)** Typical unmixing with three different bases (PpIX_634_, PpIX_620_, and CpIII), showing the residuals below, **(B)** Typical CpIII component fitting to noise in the secondary fluorescence peak.

Additionally, since this method is much more robust to noise than simply finding the maximum, the measured spectra were first up-sampled by a factor of 10 through cubic spline interpolation to obtain a more precise peak location. The resulting peak locations are given in Table 6 and Figure 5A, with similar results from both methods. Figure 5B shows the new PpIX_634_ and CpIII spectra obtained from the phantom measurements. Both PpIX and CpIII match well with previous data from Seo et al. (2009), as seen in Figure 6. These observations show that CpIII is similar to the previously published basis spectrum of PpIX_620_ (Black et al., 2021).

**Table 6:** Porphyrin peak fluorescence locations from the two methods.

Substance	Peak Location (from measured max)	Peak Location (from fit)
CpIII	616.7 ± 0.6 nm	616.8 ± 0.0 nm
PpIX	634.7 ± 0.3 nm	634.7 ± 0.0 nm

We performed unmixing experiments using the average spectra of the individual fluorophore phantoms as new basis spectra, shown in Figure 5B. We used three different sets of basis spectra: A = [PpIX_634_, PpIX_620_, autofluorescence], B = [PpIX_634_, CpIII, autofluorescence], and C = [PpIX_634_, PpIX_620_, CpIII, autofluorescence], where the autofluorescence was lipofuscin, flavin, and NADH (Black et al., 2021).

First, pig brain phantoms spiked with PpIX and CpIII were unmixed (1995 spectra). Basis C gave a significantly better unmixing result than basis B and A (*p* < 0.001), with root mean square (RMS) unmixing errors (A = 0.0038, B = 0.0029, and C = 0.0026). This suggests that neither CpIII nor PpIX_620_ alone are sufficient to describe the spectral characteristics of pig brain phantoms and that a model like set C, which uses three porphyrin spectra (PpIX_634_, PpIX_620,_ and CpIII), is superior. Alternatively, the better performance of set C could be due to slight overfitting.

Next, the human brain spectra were unmixed. The RMS unmixing error of A and C were significantly lower than B (*p* < 0.001), again indicating that CpIII alone does not effectively account for PpIX_620_. The average ± standard deviation abundances of the porphyrins from the three different unmixing processes are shown in Table 7. Note that each basis spectrum was initially normalized to have the same area under the curve, so the abundances can be compared directly, though they are in arbitrary units.

**Table 7:** Abundances of potential porphyrins in human brain spectra, calculated by fitting the human brain spectra with specific portions of the basis spectra shown in Figure 5B and autofluorescence (lipofuscin, flavin, NADH) as described previously (Black et al., 2021).

A	[PpIX_634_]	[PpIX_620_]	
RMS error: 0.0046 ± 0.0073	3.98 ± 8.68	0.71 ± 1.68	
B	[PpIX_634_]		[CpIII]
RMS error: 0.0052 ± 0.0086	4.02 ± 8.79		0.59 ± 1.45
C	[PpIX_634_]	[PpIX_620_]	[CpIII]
RMS error: 0.0046 ± 0.0073	3.97 ± 8.69	0.68 ± 1.68	0.049 ± 0.17

The abundance of PpIX_634_ and PpIX_620_ using set A and C were similar, and the published PpIX_620_ basis spectrum (Black et al., 2021) alone performs better than that of CpIII. The difference may be because the PpIX_620_ spectrum is more optimized for human data, whereas the CpIII spectrum was derived from spiked pig brain experiments and was not naturally occurring. We also used a PpIX spectrum, shifted to peak at 616 nm, as an approximation for Up and found its abundance negligible (< 0.01) in all cases. This implies that Up is probably not commonly present in the measured human spectra, though we have not carefully characterized its spectrum.

It is also possible to compare the unmixing process qualitatively. The fits of set A versus B in human data were visibly different, with set A using the PpIX_620_ (Black et al., 2021) basis spectrum instead of the CpIII spectrum, giving a better fit around the two peaks. Using set C does improve the fit slightly, possibly due to overfitting. A typical unmixing with set A, B, and C is shown in Figure 6A. In measurements where a non-negligible CpIII component is fitted, the secondary peak is often noisy, so CpIII is fitted to boost up this portion of the fitted spectrum. It usually results in a fit where the primary peak is overly blue-shifted due to the location of the primary peak of CpIII. A typical example is shown in Figure 6B.

The difference in fit error between basis A and C is minimal in all unmixing. The combination of all three spectra may work best because, with three spectra, we are overfitting. Indeed, in the human data, the RMS fit errors of basis C and A are identical, at 0.0046 ± 0.0073 (mean ± standard deviation). This is because the attributed CpIII abundance is negligible. The [CpIII] is 14 times smaller than that of [PpIX_620_] and 80 times smaller than that of [PpIX_634_].

This suggests that either (1) CpIII and the peak so far labeled as PpIX_620_ originate from the same fluorophore, where the difference in shape stems from the different measurement conditions (human tumor versus spiked pig brain), or (2) CpIII is not present in appreciable abundances in the human measurements, and PpIX_620_ is probably a state of PpIX. It is challenging to distinguish conclusively between PpIX_620_ and CpIII in the spectra as both are very similar. The basis vectors in the unmixing should be as mutually orthogonal as possible, but CpIII and PpIX_620_ are relatively colinear due to their similarity. Hence, the unmixing problem with basis C becomes more degenerate, and different values of [CpIII] and [PpIX_620_] can give almost equally good results. Due to the degeneracy, overfitting, and the two possible conclusions above, we postulate that one should use only the PpIX_620_ basis spectrum in the unmixing.

On the other hand, Up is not appreciably present in human tumor data. Besides Up, Cp, and PpIX, there are also hepta-, hexa- and penta-carboxylporphyrinogens, which are intermediates in heme biosynthesis between Up and CpIII. These porphyrins should be kept in mind for future projects.

### Tumor types

**Figure 7:**
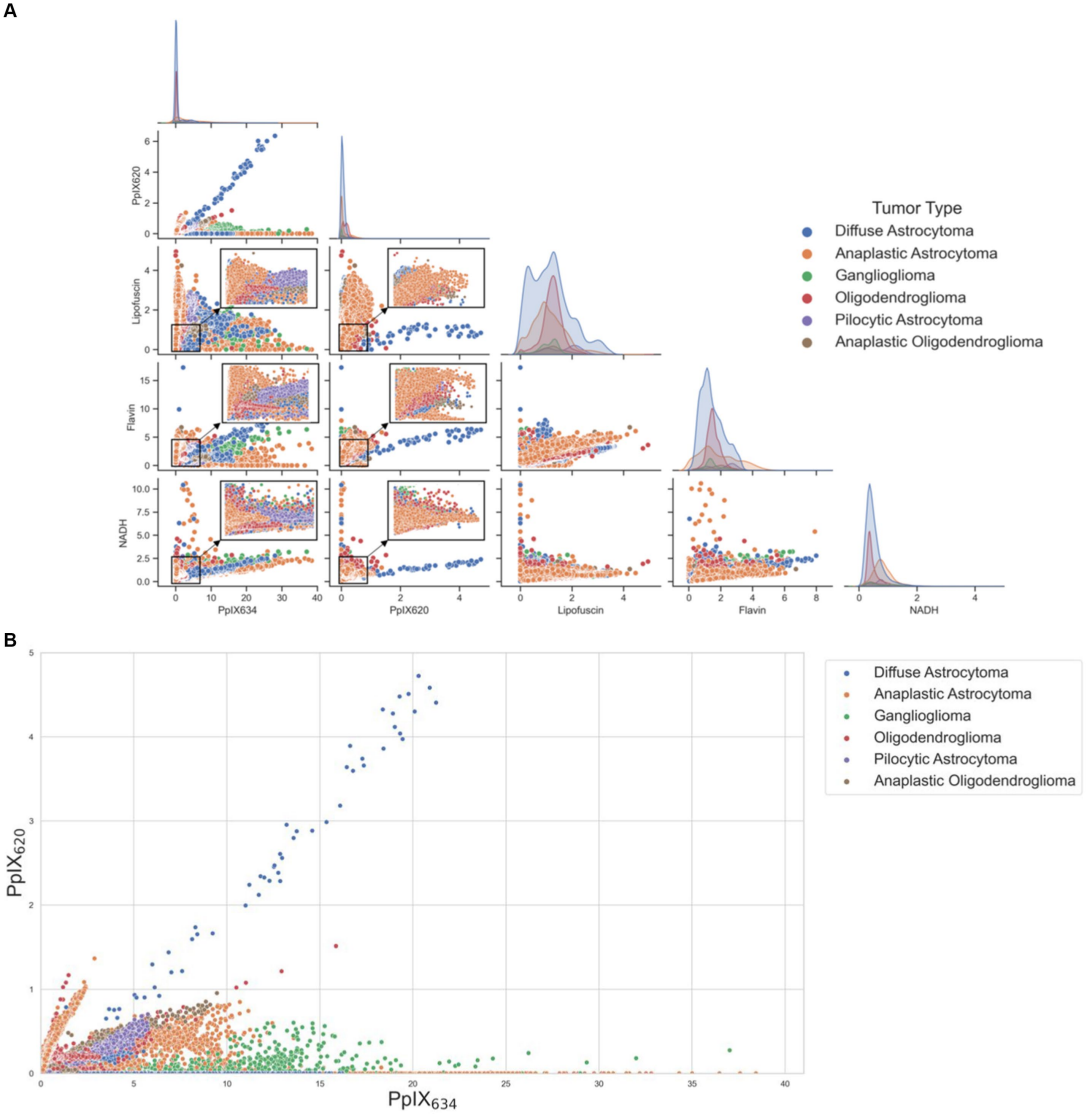
**(A)** Scatterplot matrix demonstrating cluster formation within tumor types when divided by the five basis spectra. Clusters within the scatterplot were magnified, emphasizing regions of higher data density. The diagonal shows the distribution of the respective abundances of the basis spectra for each tumor subtype. **(B)** Scatterplot of different tumor types and their distribution among [PpIX_620_] and [PpIX_634_].

Differences in spectral characteristics between different tumor types were analyzed with the five basis spectra (PpIX_634_, PpIX_620_, lipofuscin, flavin, NADH) (Black et al., 2021). The differences in [PpIX_634_] and [PpIX_620_] between tumor types were statistically significant (*p* < 0.01), but again, [PpIX_634_] seems to vary much more than [PpIX_620_] between the different classes. By plotting the five basis spectra among various tumor entities (Figure 7A), clusters can be built, indicating different abundances of autofluorescence according to the tumor type. Figure 7B shows the distribution among [PpIX_620_] and [PpIX_634_] for different tumor types.

## Discussion

### Significance of the 620 nm fluorescence

The origin of the 620 nm fluorescence peak, which is blue-shifted compared to the well-known PpIX fluorescence maximum at 634 nm, is of ongoing discussion. Previous work suggested that the signal arose from a second PpIX photo-state (Montcel et al., 2013; Alston et al., 2019; Black et al., 2021). The reason for this was data that showed the appearance of a fluorescence maximum at 620 nm in an aqueous solution containing only PpIX and dependent on the physicochemical aggregation behavior of PpIX (Melo and Reisaeter, 1986; Scolaro et al., 2002). It was, thus, for a good reason, assumed that the signal at 620 nm in neurosurgical applications is a second photo-state of PpIX and that the PpIX photo-state distribution is dependent on the microenvironment as this affects PpIX aggregation. Consequently, aqueous phantoms were developed, which proved the observations of Melo and Reisaeter (Melo and Reisaeter, 1986). Shifting the PpIX fluorescence maximum between 634 and 620 nm was possible by variation of surfactant, lipid volume fraction, and pH (Alston et al., 2018).

Moreover, addition of human serum albumin (Lozovaya et al., 1990), or isolated proteinoid solution (Lozovaya et al., 1990), also shifted the PpIX fluorescence from 620 nm in aqueous solution, which is attributed to a highly aggregated PpIX micelle (Melo and Reisaeter, 1986), towards 634 nm, an effect commonly observed in organic solvents like methanol (Lozovaya et al., 1990). During our experiments with pH-RTHs, the pH did not affect the 620 nm fluorescence much, possibly because the aggregation behavior of PpIX was different in pH-RTHs compared to aqueous phantoms (Alston et al., 2018). Thus, in aqueous solutions and phantoms, the published data and our results imply that (i) both fluorescence maxima originated from PpIX, as this was the only fluorophore present, (ii) the appearance of different PpIX fluorescence emission maxima was foremost due to the PpIX aggregation behavior, (iii) the aggregation behavior was dependent on the microenvironment in aqueous solutions and phantoms, which is different from RTHs. Still, it is unclear if these observations can be transferred to an *in vivo* setting, where, most likely, aggregation of PpIX is prevented by proteins, e.g., albumin (Lamola et al., 1981), and association of PpIX to lipid bilayers like the cell or mitochondria membrane.

In a biological system, if the heme biosynthesis is performed *in situ*, it becomes more reasonable that the 620 nm fluorescence emission could be attributed to other porphyrin precursors like Up or CpIII. Indeed, the 620 nm signal was labeled as a more hydrophilic PpIX precursor during spectral studies in the human skin (Seo et al., 2009), blood plasma (Lang et al., 2015), bacteria cultures of the human digestive tract (Dietel et al., 2007), or in carcinoma cells (Dietel et al., 1996). In some instances, the spectral results were verified using high-performance liquid chromatography (HPLC) (Dietel et al., 1996), which is more specific than spectral fluorescence. Dietel and co-workers identified Up, Cp, and traces of hepta-, hexa- and penta-carboxyporphyrin besides PpIX in carcinoma cells after induction of heme biosynthesis with 5-ALA (Dietel et al., 1996).

**Figure 8:**
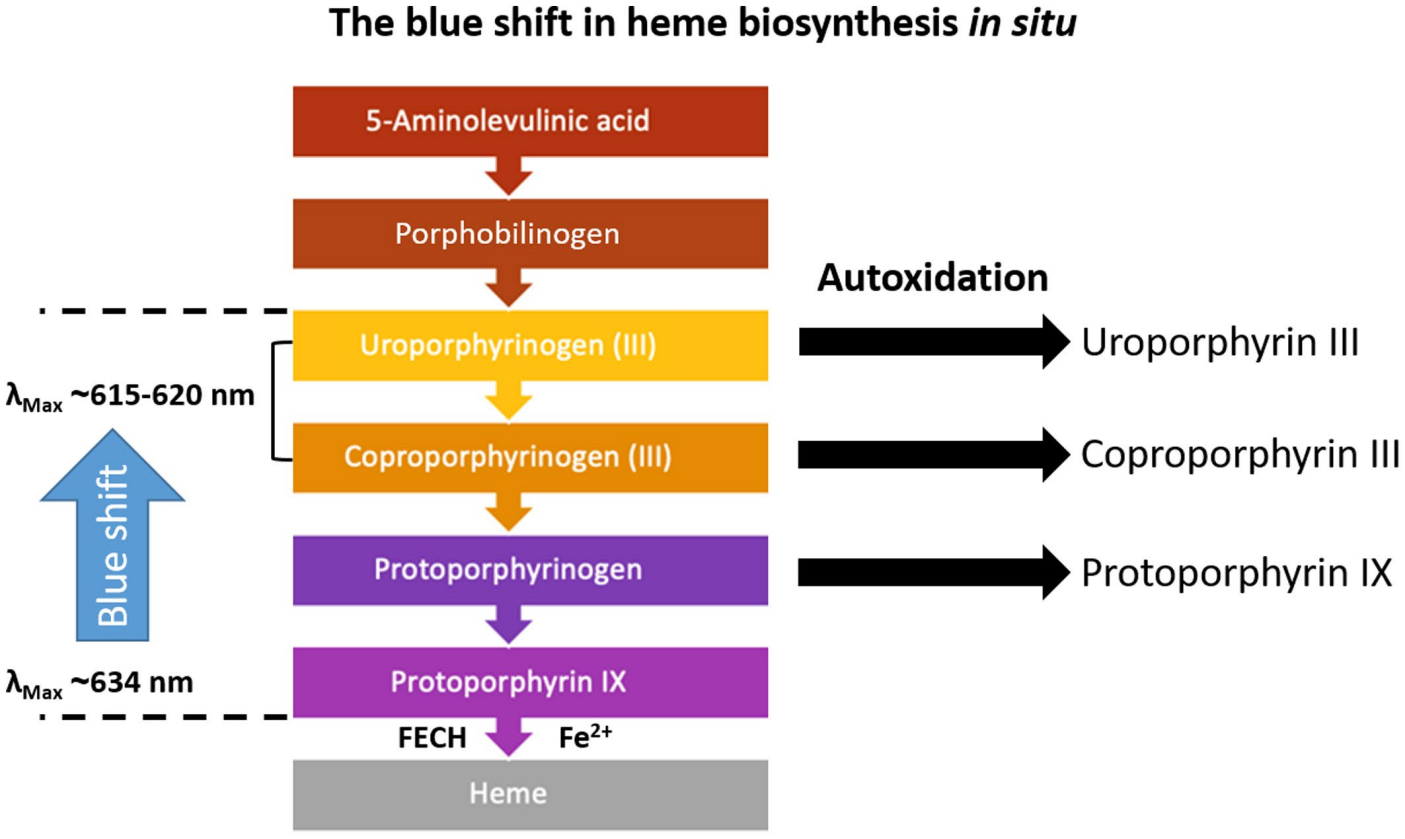
Heme biosynthesis and fluorescence peak maxima according to the literature (Dietel et al., 2007; Seo et al., 2009; Lang et al., 2015). The porphyrinogen intermediates do not fluoresce, but they readily oxidize *in situ*, if oxygen is available, and form the delocalized system of π-electrons, which facilitates fluorescence. This process is further enhanced by irradiation.

The exact mechanisms that lead to induction of heme biosynthesis and accumulation of PpIX in glioma tissue are multifactorial and not entirely clear (Stepp and Stummer, 2018; McNicholas et al., 2019). After 5-ALA enters the cell, the tumor microenvironment, e.g., hypoxia due to altered glucose metabolism, may play an important role, as well as alterations in heme biosynthesis (McNicholas et al., 2019). Often, reduced activity of the enzyme ferrochelatase (FECH) and iron deficiency are proposed to be linked to the PpIX accumulation in cancer cells (Valdes et al., 2010; Stepp and Stummer, 2018; McNicholas et al., 2019). Still, evidence is missing that this plays a role *in vivo*. Nevertheless, synthesis of PpIX is only possible via induction of the whole heme synthesis pathway, including the more hydrophilic porphyrin precursors. Closely linked to heme biosynthesis is a group of genetic metabolic disorders, the porphyrias. There, dysfunction in any of the enzymes in the heme pathway can lead to accumulation of a heme precursor (Figure 8) in blood, urine, and stool (Bissell and Wang, 2015; Lang et al., 2015; Bissell et al., 2017). It is, therefore, possible that the more hydrophilic porphyrins are also elevated in cancer besides the well-known substantial accumulation of PpIX. Future work should aim to characterize the porphyrin pattern in cancer by spectral analysis, accompanied by more specific analytical methods, e.g., HPLC and mass spectrometry. Also, an excitation at 405 nm might account for a low quantum yield of PpIX_620_/CpIII. Additionally, heme biosynthesis enzymes should be included in these studies to understand the cause of porphyrin accumulation. Such studies would also contribute to elucidating the mechanisms that lead to PpIX accumulation in glioma cells.

### The blue shift

In previous work, while characterizing [PpIX_634_] and [PpIX_620_] and calculating the proposed ratio, a shift in the measured spectrum from 634 nm to 620 nm was observed. This blue shift was progressive with decreasing malignancy and was significantly correlated with [PpIX_634_], [PpIX_620_], Ki-67 proliferation index, fluorescence visibility, and WHO grade of tumors (Black et al., 2021). [PpIX_620_] is artificially inflated when not unmixing with sufficient basis spectra; the blue shift was proposed as a more robust biomarker than the PpIX_620/634_ ratio, as it is independent of the autofluorescence (Black et al., 2021).

According to our analysis, [PpIX_634_] is much more informative for all the assessed classifications than [PpIX_620_]. Therefore, it is sufficient to note this abundance as the main one regarding optical device development. [PpIX_620_] does not vary much with pH, while [PpIX_634_] changes approximately linearly. The same is true for IDH mutation and fluorescence visibility. In both cases, [PpIX_634_] strongly correlates with the respective variable, whereas [PpIX_620_] is relatively constant. The ratio [PpIX_620_]/[PpIX_634_] is significantly greater for IDH-mutant tumors than IDH-wildtype and for non-fluorescing tumors greater than strongly fluorescing ones. [PpIX_634_] also varies much more than [PpIX_620_] between different tumor types. Still, in general, other tumor types seem to have different amounts of lipofuscin, NADH, flavin, PpIX_620_, and PpIX_634_ fluorescence (Figure 7). Still, again, it is mostly [PpIX_634_] that changes. Doubling the dose of 5-ALA seems to decrease the proportion of [PpIX_620_]. Both [PpIX_620_] and [PpIX_634_] vary approximately by the same amount when assessing the tumor margins between ST, IZ, and RABT. As a result, the ratio is approximately constant.

As a potential limitation of this study, it has to be noted that experiments were performed *ex vivo* with a wide-field HI device, and previously described work about the two-peaked 5-ALA-induced PpIX fluorescence emission spectrum was described *in vivo* by using a different technique from HI, i.e., fiber optic point spectroscopy (Montcel et al., 2013; Alston et al., 2019). Therefore, results might not be entirely comparable. However, this study measured *ex vivo* tissue immediately after extraction. We still believe our analysis in a large collective demonstrates the importance of the unmixing method with the appropriate basis spectra (Black et al., 2021), as we still see artificially inflated [PpIX_620_] when not correctly unmixing.

## Conclusion

[PpIX_634_] mainly contributes to visual 5-ALA-induced fluorescence in brain tumors. [PpIX_620_] varied only slightly with pH and different tumor regions, IDH status, and fluorescence visibility. Other porphyrin intermediates of heme biosynthesis are discussed as a possible origin of the blue-shifted 620 nm fluorescence emission. One potential candidate, CpIII, was examined here, revealing similar spectral characteristics to PpIX_620_. Given the spectral complexity of the measurements, the contribution of multiple fluorophores, the value of information contained in each fluorophore, and the risk of drawing incorrect conclusions on the identity of fluorophores, it is important to continue exploring the spectra and identifying their sources. Together with improved spectral unmixing methods, this will contribute to a deeper understanding of tumor biology.

## Data availability statement

The datasets presented in this article are not readily available because these large database of biopsies has not been approved for sharing publicly. Requests to access the datasets should be directed to eric.suero@ukmuenster.de.

## Ethics statement

The studies involving humans were approved by the Ethics committee, University of Münster. The studies were conducted in accordance with the local legislation and institutional requirements. Written informed consent for participation in this study was provided by the participants’ legal guardians/next of kin. The animal studies were approved by Health and Veterinary Office Münster. The studies were conducted in accordance with the local legislation and institutional requirements. Written informed consent was not obtained from the owners for the participation of their animals in this study because pig brain was acquired from a local butcher.

## Author contributions

EM: Conceptualization, Data curation, Formal analysis, Investigation, Methodology, Software, Supervision, Validation, Visualization, Writing – original draft, Writing – review & editing. DB: Conceptualization, Data curation, Formal analysis, Methodology, Visualization, Writing – original draft, Writing – review & editing. AW: Investigation, Methodology, Writing – original draft, Writing – review & editing, Data curation. GA: Software, Visualization, Writing – review & editing. Fd’A: Investigation, Methodology, Writing – review & editing. SK: Investigation, Methodology, Project administration, Supervision, Writing – review & editing. WS: Investigation, Project administration, Supervision, Writing – review & editing.

## References

[ref1] Alston L. Mahieu-Williame L. Hebert M. Kantapareddy P. Meyronet D. Rousseau D.. (2019). Spectral complexity of 5-ala induced Ppix fluorescence in guided surgery: a clinical study towards the discrimination of healthy tissue and margin boundaries in high and low grade gliomas. Biomed. Opt. Express 10, 2478–2492. doi: 10.1364/BOE.10.002478, PMID: 31149380 PMC6524587

[ref2] Alston L. Rousseau D. Hebert M. Mahieu-Williame L. Montcel B. (2018). Nonlinear relation between concentration and fluorescence emission of Protoporphyrin ix in calibrated phantoms. J. Biomed. Opt. 23, 1–7. doi: 10.1117/1.JBO.23.9.097002, PMID: 30251489

[ref3] Bissell D. M. Anderson K. E. Bonkovsky H. L. (2017). Porphyria. N. Engl. J. Med. 377:2101. 10.1056/NEJMc1712682 29166231

[ref4] Bissell D. M. Wang B. (2015). Acute hepatic Porphyria. J. Clin. Transl. Hepatol. 3, 17–26. doi: 10.14218/JCTH.2014.00039, PMID: 26357631 PMC4542079

[ref5] Black D. Kaneko S. Walke A. Konig S. Stummer W. Suero Molina E. (2021). Characterization of autofluorescence and quantitative Protoporphyrin ix biomarkers for optical spectroscopy-guided glioma surgery. Sci. Rep. 11:20009. doi: 10.1038/s41598-021-99228-6, PMID: 34625597 PMC8501114

[ref6] Bravo J. J. Olson J. D. Davis S. C. Roberts D. W. Paulsen K. D. Kanick S. C. (2017). Hyperspectral data processing improves Ppix contrast during fluorescence guided surgery of human brain tumors. Sci. Rep. 7:9455. doi: 10.1038/s41598-017-09727-8, PMID: 28842674 PMC5572708

[ref7] Dietel W. Bolsen K. Dickson E. Fritsch C. Pottier R. Wendenburg R. (1996). Formation of water-soluble porphyrins and Protoporphyrin ix in 5-Aminolevulinic-acid-incubated carcinoma cells. J. Photochem. Photobiol. B 33, 225–231. doi: 10.1016/1011-1344(95)07249-7, PMID: 8683398

[ref8] Dietel W. Pottier R. Pfister W. Schleier P. Zinner K. (2007). 5-Aminolaevulinic acid (ala) induced formation of different fluorescent porphyrins: a study of the biosynthesis of porphyrins by Bacteria of the human digestive tract. J. Photochem. Photobiol. B 86, 77–86. doi: 10.1016/j.jphotobiol.2006.07.006 16973372

[ref9] Gerweck L. E. Seetharaman K. (1996). Cellular Ph gradient in tumor versus Normal tissue: potential exploitation for the treatment of Cancer. Cancer Res. 56, 1194–1198. PMID: 8640796

[ref10] Honasoge A. Sontheimer H. (2013). Involvement of tumor acidification in brain Cancer pathophysiology. Front. Physiol. 4:316. doi: 10.3389/fphys.2013.00316 24198789 PMC3814515

[ref11] Jaber M. Wolfer J. Ewelt C. Holling M. Hasselblatt M. Niederstadt T.. (2016). The value of 5-Aminolevulinic acid in low-grade gliomas and high-grade gliomas lacking glioblastoma imaging features: an analysis based on fluorescence, magnetic resonance imaging, 18f-Fluoroethyl tyrosine positron emission tomography, and tumor molecular factors. Neurosurgery 78, 401–411; Discussion 411. doi: 10.1227/NEU.0000000000001020, PMID: 26366972 PMC4747980

[ref12] Jonin C. Ray C. Salmon E. Leclerc P. Montcel B. Mahieu-Williame L.. (2020). Two photon excited fluorescence and hyper Rayleigh scattering of Protoporphyrin ix. J. Photochem. Photobiol. A Chem. 402:112812. doi: 10.1016/j.jphotochem.2020.112812

[ref13] Kaneko S. Suero Molina E. Ewelt C. Warneke N. Stummer W. (2019). Fluorescence-based measurement of real-time kinetics of Protoporphyrin ix after 5-Aminolevulinic acid administration in human in situ malignant gliomas. Neurosurgery 85, E739–E746. doi: 10.1093/neuros/nyz129, PMID: 31058995

[ref14] Kaneko S. Suero Molina E. Sporns P. Schipmann S. Black D. Stummer W. (2021). Fluorescence real-time kinetics of Protoporphyrin ix after 5-ala Administration in low-grade glioma. J. Neurosurg. 136, 1–7. doi: 10.3171/2020.10.JNS202881, PMID: 34144512

[ref15] Kim A. Khurana M. Moriyama Y. Wilson B. C. (2010). Quantification of in vivo fluorescence decoupled from the effects of tissue optical properties using Fiber-optic spectroscopy measurements. J. Biomed. Opt. 15:067006. doi: 10.1117/1.3523616, PMID: 21198210 PMC3025598

[ref16] Lamola A. A. Asher I. Muller-Eberhard U. Poh-Fitzpatrick M. (1981). Fluorimetric study of the binding of Protoporphyrin to Haemopexin and albumin. Biochem. J. 196, 693–698. doi: 10.1042/bj1960693, PMID: 7317009 PMC1163087

[ref17] Lang A. Stepp H. Homann C. Hennig G. Brittenham G. M. Vogeser M. (2015). Rapid screening test for Porphyria diagnosis using fluorescence spectroscopy. Clin. Biomed. Spectrosc. Imag. 9537

[ref18] Lozovaya G. I. Masinovsky Z. Sivash A. A. (1990). Protoporphyrin ix as a possible ancient photosensitizer: spectral and photochemical studies. Orig. Life Evol. Biosph. 20, 321–330. doi: 10.1007/BF01808114

[ref19] Mangotra H. Srivastava S. Jaiswal G. Rani R. Sharma A. (2023). Hyperspectral imaging for early diagnosis of diseases: a review. Expert. Syst. 40. doi: 10.1111/exsy.13311

[ref20] Mcnicholas K. Macgregor M. N. Gleadle J. M. (2019). In order for the light to Shine so brightly, the darkness must be present-why do cancers fluoresce with 5-Aminolaevulinic acid? Br. J. Cancer 121, 631–639. doi: 10.1038/s41416-019-0516-4 31406300 PMC6889380

[ref21] Melo T. B. Reisaeter G. (1986). The physicochemical state of Protoporphyrin ix in aqueous solution investigated by fluorescence and light scattering. Biophys. Chem. 25, 99–104. doi: 10.1016/0301-4622(86)85070-0 17010276

[ref22] Montcel B. Mahieu-Williame L. Armoiry X. Meyronet D. Guyotat J. (2013). Two-peaked 5-ala-induced Ppix fluorescence emission Spectrum distinguishes glioblastomas from low grade gliomas and infiltrative component of glioblastomas. Biomed. Opt. Express 4, 548–558. doi: 10.1364/BOE.4.000548, PMID: 23577290 PMC3617717

[ref23] Petrecca K. Guiot M. C. Panet-Raymond V. Souhami L. (2013). Failure pattern following complete resection plus radiotherapy and Temozolomide is at the resection margin in patients with glioblastoma. J. Neuro-Oncol. 111, 19–23. doi: 10.1007/s11060-012-0983-4 23054563

[ref24] Schaff L. R. Mellinghoff I. K. (2023). Glioblastoma and other primary brain malignancies in adults: a review. JAMA 329, 574–587. doi: 10.1001/jama.2023.0023 36809318 PMC11445779

[ref25] Schipmann S. Muther M. Stogbauer L. Zimmer S. Brokinkel B. Holling M.. (2020). Combination of ala-induced fluorescence-guided resection and intraoperative open photodynamic therapy for recurrent glioblastoma: case series on a promising dual strategy for local tumor control. J. Neurosurg. 134, 1–11. 10.3171/2019.11.JNS192443 31978877

[ref26] Schucht P. Knittel S. Slotboom J. Seidel K. Murek M. Jilch A.. (2014). 5-ala complete resections go beyond Mr contrast enhancement: shift corrected volumetric analysis of the extent of resection in surgery for glioblastoma. Acta Neurochir. 156, 305–312; Discussion 312. doi: 10.1007/s00701-013-1906-7 24449075

[ref27] Scolaro L. M. Castriciano M. Romeo A. Patanè S. Cefalì E. Allegrini M. (2002). Aggregation behavior of Protoporphyrin ix in aqueous solutions: clear evidence of vesicle formation. J. Phys. Chem. B 106, 2453–2459. doi: 10.1021/jp013155h

[ref28] Seo I. Tseng S. H. Cula G. O. Bargo P. R. Kollias N. (2009). Fluorescence spectroscopy for endogenous porphyrins in human facial skin. Photo. Therap. Diagn. V:7161.

[ref29] Stepp H. Stummer W. (2018). 5-ala in the management of malignant glioma. Lasers Surg. Med. 50, 399–419. doi: 10.1002/lsm.22933 29737540

[ref30] Stummer W. Pichlmeier U. Meinel T. Wiestler O. D. Zanella F. Reulen H. J.. (2006). Fluorescence-guided surgery with 5-Aminolevulinic acid for resection of malignant glioma: a randomised controlled multicentre phase iii trial. Lancet Oncol. 7, 392–401. doi: 10.1016/S1470-2045(06)70665-9 16648043

[ref31] Stummer W. Stepp H. Moller G. Ehrhardt A. Leonhard M. Reulen H. J. (1998). Technical principles for Protoporphyrin-ix-fluorescence guided microsurgical resection of malignant glioma tissue. Acta Neurochir. 140, 995–1000. doi: 10.1007/s007010050206, PMID: 9856241

[ref32] Stummer W. Stepp H. Wiestler O. D. Pichlmeier U. (2017). Randomized, prospective double-blinded study comparing 3 different doses of 5-Aminolevulinic acid for fluorescence-guided resections of malignant gliomas. Neurosurgery 81, 230–239. doi: 10.1093/neuros/nyx074 28379547 PMC5808499

[ref33] Stummer W. Suero Molina E. (2017). Fluorescence imaging/agents in tumor resection. Neurosurg. Clin. N. Am. 28, 569–583. doi: 10.1016/j.nec.2017.05.009 28917285

[ref34] Suero Molina E. Black D. Kaneko S. Muther M. Stummer W. (2022). Double dose of 5-Aminolevulinic acid and its effect on Protoporphyrin ix accumulation in low-grade glioma. J. Neurosurg. 134, 1–10. 10.3171/2021.12.JNS211724 35213830

[ref35] Suero Molina E. Kaneko S. Black D. Stummer W. (2021). 5-Aminolevulinic acid-induced porphyrin contents in various brain tumors: implications regarding imaging device design and their validation. Neurosurgery 89, 1132–1140. doi: 10.1093/neuros/nyab361, PMID: 34670277

[ref36] Suero Molina E. Stogbauer L. Jeibmann A. Warneke N. Stummer W. (2020). Validating a new generation filter system for visualizing 5-ala-induced Ppix fluorescence in malignant glioma surgery: a proof of principle study. Acta Neurochir. 162, 785–793. doi: 10.1007/s00701-020-04227-7, PMID: 32034493 PMC7066295

[ref37] Valdes P. A. Angelo J. P. Choi H. S. Gioux S. (2017). Qf-Ssop: real-time optical property corrected fluorescence imaging. Biomed. Opt. Express 8, 3597–3605. doi: 10.1364/BOE.8.003597, PMID: 28856038 PMC5560828

[ref38] Valdes P. A. Leblond F. Kim A. Wilson B. C. Paulsen K. D. Roberts D. W. (2012). A spectrally constrained dual-band normalization technique for Protoporphyrin ix quantification in fluorescence-guided surgery. Opt. Lett. 37, 1817–1819. doi: 10.1364/OL.37.001817, PMID: 22660039 PMC3774026

[ref39] Valdes P. A. Samkoe K. O'hara J. A. Roberts D. W. Paulsen K. D. Pogue B. W. (2010). Deferoxamine Iron chelation increases Delta-Aminolevulinic acid induced Protoporphyrin ix in xenograft glioma model. Photochem. Photobiol. 86, 471–475. doi: 10.1111/j.1751-1097.2009.00664.x, PMID: 20003159 PMC2875336

[ref40] Walke A. Black D. Valdes P. A. Stummer W. Konig S. Suero-Molina E. (2023). Challenges in, and recommendations for, hyperspectral imaging in ex vivo malignant glioma biopsy measurements. Sci. Rep. 13:3829. doi: 10.1038/s41598-023-30680-2, PMID: 36882505 PMC9992662

